# Predictive Ability of the Classification of Ground Level Falls As Syncopal Versus Mechanical in a Mixed Intensive Care Unit Population: A Retrospective Cohort Study

**DOI:** 10.7759/cureus.88180

**Published:** 2025-07-17

**Authors:** John Culhane, Raymond Okeke, Emily Ta, Mir Saleem

**Affiliations:** 1 Surgery, Saint Louis University, St. Louis, USA; 2 Surgery, Burrell College of Osteopathic Medicine, Melbourne, USA; 3 Anatomy, Burrell College of Osteopathic Medicine, Melbourne, USA

**Keywords:** elderly falls, geriatric injuries, mechanical falls, non-fatal geriatric falls, syncopal episodes

## Abstract

Introduction

Ground-level falls are a common mechanism of trauma, especially in the elderly. They are typically classified by etiology as syncopal or mechanical, based on a report of syncope (loss of consciousness due to transient reduction of cerebral blood flow). Syncopal falls generally prompt an evaluation of the cause of syncope, but the utility of this investigation and hence this classification system remains uncertain.

Methods

This retrospective registry review compares outcomes for patients with falls classified as syncopal versus mechanical. The data source is the Medical Information Mart for Intensive Care (MIMIC) III database. Patients experiencing a fall documented in the history of present illness (HPI) or during the admission were included. Syncopal versus mechanical etiology was analyzed as a predictive factor for length of stay, mortality, and cardiovascular and neurologic outcomes. Significance for categorical variables was tested with a chi-square and continuous variables with a T-test. Multivariate analysis was performed with logistic regression for binary outcomes and linear regression for continuous outcomes.

Results

Overall mortality for mechanical falls was 384 (54.2%) versus 480 (45.2%) for syncopal (p<0.001). Conditions more common among the syncopal group include cardiac valve disorder - 151 (14.2%) versus 76 (10.7%) (p=0.038), arrhythmia - 540 (50.8%) versus 322 (45.5%) (p=0.03), orthostatic hypotension - 38 (3.6%) versus 8 (1.1%) (p=0.003), and need for pacemaker implant or revision - 87 (8.2%) versus 13 (1.8%) (p<0.001). The difference in myocardial infarction was not significant. Syncopal etiology was an independent predictor of outcomes including overall mortality - adjusted odds ratio (OR) 0.75 (p=0.005), gastrointestinal bleed- (OR) 1.74 (p=<0.001), orthostatic hypotension - OR 3.34 (p=0.002), and need for pacemaker placement or revision - OR 4.0 (p<0.001).

Conclusion

Mortality was lower for patients with syncopal falls. Cardiovascular conditions were significantly more common among syncopal fall patients, but the incidence, especially for arrhythmia, was high and nearly equal in the mechanical group. We believe that a standard workup for orthostasis and arrhythmia should be performed for both groups, regardless of syncopal versus mechanical etiology.

## Introduction

Ground-level falls are a common mechanism of trauma, especially in the elderly. Approximately one-third of adults aged 65 years or older fall per year, resulting in nearly 2.3 million non-fatal falls in 2010 [1]. Ground-level falls are typically classified as syncopal or mechanical. A syncopal event is defined as a loss of consciousness caused by a transient reduction of cerebral blood flow [2]. When this occurs in an upright posture, the result is a syncopal fall. A mechanical fall, by contrast, is due to the application of external force or environmental contact, such as walking into an object, slipping on ice, or tripping over a carpet. The event may also be related to patient characteristics such as muscle weakness or vision loss [3].

A fall classified as syncopal generally prompts a syncopal evaluation. Syncope is commonly divided into cardiac and non-cardiac etiology. Cardiac syncope is considered a greater threat to life; hence, cardiac conditions are the focus of testing [4]. Evaluation for cardiac syncope frequently includes a 12-lead electrocardiogram (ECG), telemetry, and echocardiogram. Sometimes troponins are ordered to screen for laboratory evidence of myocardial infarction (MI). Neurologic causes of sudden loss of consciousness, while not technically syncope, may also result in falls. Concern for neurologic causes may prompt neurologic tests such as brain imaging and electroencephalogram (EEG) [4].

There is controversy regarding the threshold and extent of workup for syncope in general, regardless of whether it is associated with a fall. Most agree that a screening ECG is necessary, but there is still disagreement and practice variation related to the need for admission, telemetry, neurologic imaging, and cardiac ischemic workup. Furthermore, syncope guidelines are not externally validated in the trauma setting [5]. Some previous studies have shown that universal testing is low yield [6, 7], while others have documented changes in evaluation and treatment based on syncopal investigation [3].

This study evaluates ground-level fall patients in the intensive care unit (ICU) setting, which gives us a much older and sicker cohort. Adverse outcomes are more common than in a typical group of emergency room (ER) patients selected for a chief complaint of falls. While the exclusively ICU population limits our ability to generalize to all falls, it gives us more power to detect a difference in the clinical courses of the two types of falls. The population also provides two well-matched cohorts that differ only by the incidence of syncope. By comparing the treatment course of these two groups, the authors hope to gain an understanding that will prompt further study to extrapolate these results from the ICU population to syncopal patients in other contexts.

Our objectives are to compare the frequency of selected cardiovascular and neurologic findings in patients with falls explicitly labeled as syncopal versus mechanical in the medical record. If these conditions are found with a meaningfully greater frequency in one group versus the other, providers may approach this group with a higher index of suspicion and a lower threshold to conduct a workup. If the syncopal versus mechanical classification fails to predict important differences in the clinical course, we would conclude that this classification system is less useful. The hypothesis is that cardiovascular and neurologic diagnoses and procedures are more common for patients who have a syncopal rather than a mechanical fall.

## Materials and methods

This is a retrospective registry review that compares outcomes for patients with falls classified as syncopal versus mechanical. The data source is the Medical Information Mart for Intensive Care (MIMIC) III database. MIMIC is a public registry of ICU data from Beth Israel Deaconess Hospital in Boston. While not the most recent version of this database, MIMIC III was chosen because it includes all daily progress notes. Patients are selected from a mixed medical and surgical population, which includes trauma.

Inclusion criteria included all patients with a documented history of a syncopal or mechanical fall. The admissions extend over 11 years from 2001 through 2012. All available records were examined. The final sample size was determined by the number of patients with falls explicitly classified as syncopal versus mechanical. Ages 17 through 90+ were included. MIMIC groups together all ages greater than 90 as part of their de-identification algorithm. Exclusion criteria are age under 17 and insufficient information to classify the fall. All patients in the data set meeting the criteria were included in the study. The sample size was determined by the number of patients who met these criteria.

Free text notes were examined using software developed at our institution. The history of present illness, discharge summary, and all progress notes were reviewed for any patient experiencing a fall as part of the history of present illness (HPI) or during the fall-associated admission. A history of falls described in the past medical history but not the history of present illness was considered remote and not included. Falls were included only if they were explicitly categorized as syncopal or mechanical by treating providers. If there was any ambiguity, the authors analyzed the context within the note to decide. Falls described as possibly syncopal or pre-syncopal were classified as syncopal since these tend to trigger a syncopal workup. This is consistent with the recommended practice of the Expert Panels on Cardiac Imaging and Neurological Imaging [8].

Syncopal versus mechanical etiology of the fall is the independent predictor variable. Outcome variables include death, length of stay, and selected cardiac and neurologic conditions. MIMIC may include multiple admissions per patient. The only variable based on data over multiple admissions is death at any time. This was included to evaluate fall etiology as a potential marker of overall health. All other variables refer only to the index admission. Demographics relevant to our analysis were retrieved, displayed descriptively, and used as covariates in our multivariate regression. Cardiovascular and neurologic outcomes were derived from International Classification of Diseases 9 (ICD-9) codes. MIMIC III uses the 9th revision of the International Classification of Diseases, requiring us to use ICD-9 codes instead of the current ICD-10. 

Univariate analysis of categorical variables was performed with chi-square, and continuous variables with the T-test. Multivariate analysis was performed with logistic regression for binary outcomes and linear regression for continuous outcomes. Covariates were age, gender, and care unit of admission.

## Results

Baseline characteristics

Table 1 lists baseline characteristics relevant to the clinical question. The data reflect an elderly cohort with roughly even balance by gender. A history of falls was common among the ICU population. Most patients were admitted to the medical ICU. The total number of patients screened is 42561. The number of patients who experienced a fall classified as either syncopal or mechanical is 1770 (4.2% of the total). Of these, 708 (40.0%) were mechanical and 1062 (60.0%) were syncopal.

**Table 1:** Baseline Characteristics CCU (coronary care unit) and CSRU (cardiac surgery recovery unit) are the cardiac units; MICU (medical intensive care unit) includes neurology; SICU (surgical intensive care unit) includes neurosurgery; TSICU (trauma surgical intensive care unit).  P<0.001 for any difference between groups for care unit of admission. SD (standard deviation); SMD (standardized mean difference).  Syncopal and mechanical fall patients were assigned to various units based on chief complaint, associated conditions, and comorbidities. The standardized mean differences would generally be considered small for age and gender, and medium for care unit assignment

	Mechanical	Syncopal	p	SMD
Age (mean (SD))	75.97 (13.41)	72.10 (15.05)	<0.001	0.272
Gender = Male n (%)	333 (47.0)	567(53.0)	0.010	0.127
CAREUNIT n (%)			<0.001	0.435
CCU	75 (10.6)	225 (21.2)	-	-
CSRU	14 (2.0)	69 (6.5)	-	-
MICU	315 (44.5)	464 (43.7)	-	-
SICU	140 (19.8)	157 (14.8)	-	-
TSICU	164 (23.2)	147 (13.8)	-	-

Univariate analysis

Table 2 shows mortality and hospital length of stay (LOS) for patients admitted with syncopal versus mechanical falls. Mortality was common during the index admission for the fall and was extremely high over the 11 years captured by the MIMIC data set. These results show a very ill, high-mortality cohort.

**Table 2:** Mortality and hospital length of stay (LOS) for patients admitted with syncopal versus mechanical fall. Clinical outcomes for patients with mechanical versus syncopal fall. LOS: length of stay;

	Mechanical	Syncopal	p
LOS [(mean (SD)]	3.84 (5.52)	3.54 (4.48)	0.23
Death during fall-associated admission n (%)	98 (13.8)	93 (8.8)	0.001
Death at any time n (%)	384 (54.2)	480 (45.2)	<0.001

Table 3 shows selected cardiovascular and neurologic diagnoses relevant to syncope derived from International Classification of Diseases (ICD-9) codes assigned to the index admission for patients with syncopal versus mechanical fall. Cardiovascular diagnoses are extremely common, occurring in over half of the population. Most cardiovascular diagnoses consist of arrhythmia, which itself is seen in about one-half of the patients. Neurologic conditions are less common; however, traumatic brain injury occurs frequently despite the lower-force mechanism of a ground-level fall. Approximately one in five falls is described as recurrent. 

**Table 3:** Selected cardiovascular and neurologic diagnoses relevant to syncope derived from International Classification of Disease (ICD-9) codes assigned to the index admission for patients with syncopal versus mechanical fall. Selected ICD-9 codes associated with the index admission for patients with mechanical versus syncopal fall. Cardiovascular and neurologic conditions relevant to syncope were selected. Individual components of composite measures may not add up because one patient may have several diagnoses and procedures. Traumatic brain injury (TBI) is listed separately from other neurologic conditions because it is typically the result of a fall rather than an underlying condition.

	Mechanical n (%)	Syncopal n (%)	p
Cardiac valve disorder	76 (10.7)	151 (14.2)	0.038
Myocardial infarction (MI)	59 (8.3)	92 (8.7)	0.876
Arrhythmia	322 (45.5)	540 (50.8)	0.030
Orthostatic hypotension	8 (1.1)	38 (3.6)	0.003
Any cardiovascular ICD diagnosis	377 (53.2)	635 (59.8)	0.007
Seizure	27 (3.8)	42 (4.0)	0.980
Cerebrovascular accident (CVA)	32 (4.5)	62 (5.8)	0.270
Any neurologic diagnosis	58 (8.2)	98 (9.2)	0.504
Pacemaker implant or revision	13 (1.8)	87 (8.2)	<0.001
Any cardiovascular procedure	22 (3.1)	89 (8.4)	<0.001
Any cardiovascular diagnosis or procedure	379 (53.5)	646 (60.8)	0.003
Any neurologic procedure	8 (1.1)	24 (2.3)	0.117
Traumatic brain injury	170 (24.0)	184 (17.3)	0.001
Gastrointestinal bleed	61 (8.6)	153 (14.4)	<0.001
Recurrent fall	147 (20.8)	187 (17.6)	0.110

Multivariate analysis 

Syncopal etiology of fall is associated with a 0.41 day decrease in length of stay, but this result is not significant on multivariate linear regression (p=0.09).

Patients with syncopal falls had lower adjusted odds of mortality during the index admission, showing a protective association for syncopal etiology. Cardiovascular ICD-9 diagnoses and procedure codes for admissions related to syncopal versus mechanical falls are non-significant for arrhythmia, valve disorder, myocardial infarction, and the composite of all cardiovascular diagnoses. Neurologic ICD-9 diagnoses and procedure codes for admissions related to syncopal versus mechanical falls are nonsignificant. Syncopal association is significant for outcomes shown in Table 4. The strongest independent associations with syncopal mechanism are the need for a pacemaker-related procedure and orthostatic hypotension.

**Table 4:** Multivariate analysis of mortality and ICD-9 diagnoses associated with syncopal etiology of falls. OR: adjusted odds ratio.  p<0.05 is considered significant.

	OR
Death during index admission	0.63
Death at any time	0.75
Pacemaker placement or revision	4.00
Orthostatic hypotension	3.34
Gastrointestinal bleed	1.74

## Discussion

Ground-level falls are a common source of morbidity among elderly patients. About 27% of adults older than 65 suffer falls, with 10% resulting in injury. Injuries include hip and spine fractures, rib fractures causing impaired respiration, and intracranial bleeding [9]. Ground-level falls are typically classified according to their presumed etiology. The two broad categories are syncopal and mechanical. Syncopal falls occur when a transient reduction of cerebral blood flow leads to unconsciousness, whereas a mechanical fall generally results from a musculoskeletal condition or external force [4, 10]. When a fall is classified as syncopal, it typically prompts an investigation of the cause of the loss of consciousness [7]. 

Syncope falls into three categories: autonomic reflex or vasovagal (59.3%), cardiac (10.4%), and orthostatic (9.1%), with a further 11.3% unexplained [11]. Of these, cardiac represents the greatest threat to life; thus, syncopal workup focuses on evaluating cardiac disorders such as arrhythmia, valvular disease, and ischemia. Neurologic conditions such as stroke and seizures may also lead to falls, but these are not technically syncope, and are generally identified by neurologic symptoms. The workup is standard for traumatic and non-traumatic syncope. In an American Family Physician review, Coulter et al. recommend evaluation for cardiac arrhythmia and seizures for patients who lose consciousness [9]. Recurrent falls are another concern after an event provoked by syncope. Falls attributed to syncope may recur in up to 30% of patients [12].

While it seems logical to attempt to identify dangerous underlying conditions, a risk of the syncopal/mechanical fall classification may lead to over-testing for underlying conditions in falls labeled as syncopal and under-testing for those labeled as mechanical. Under-testing may miss an important diagnosis, but over-testing is also a concern because a low-yield investigation involves more than the cost of resources. False positive results may lead to a cascade of further testing and unnecessary or even dangerous treatment [13].

Various studies have questioned the practical value of routine workup for syncope associated with ground-level falls. Maung et al. identified 302 patients whose trauma was attributed to syncope. Workup overall was low-yield, with most positive results readily identifiable by symptom-based evaluation. The authors concluded that routine workup for syncopal history in trauma was not justified [6]. In a multicenter retrospective cohort study, Lee et al. showed that evaluation of falls judged to be syncopal led to more testing but few changes in therapy, suggesting that syncopal etiology is not relevant to treatment [7]. Sri-on et al. found that the syncopal/mechanical distinction did not help discriminate which patients would benefit from further workup [1].

Other authors have found evaluation based on syncope more useful. For example, Omert et al. found that 37.8% of workups prompted by a syncopal fall led to specific interventions [12]. Biffl et al. designed an algorithm recommending routine ECG and orthostatics for all syncopal falls [3]. Biffl and Sri-on also caution that the label of a mechanical fall might be used to justify a less thorough workup, which could miss important underlying conditions [1,3]. Our results support their concern. Our mechanical fall population has a very high incidence of arrhythmia that approaches that of the syncopal group, as seen in Table 3.

As an alternative to universal testing, some studies seek to identify the valuable elements of a selective workup. Harfouche et al. found an overall low yield for routine workup of syncope in trauma. The incidence of carotid pathology was similar to the population baseline. Risk factors reliably identified cardiac anomalies, demonstrating that cardiac evaluation can be safely guided by comorbidity, age, and injury severity [14]. Bhat et al. identified three risk factors for cardiac syncope in trauma: coronary artery disease, pathological Q waves, and age>65. The presence of at least one risk factor detected cardiac syncope with 100% sensitivity. Almost all cardiac pathology was detected by continuous ECG [15]. Recommendations for evaluating syncope in a non-trauma context also support the selective approach [4,10,16]. 

Several of our results may help further the current understanding. We found that arrhythmia is roughly equal and extremely common in both groups (Table 3). Much of this may represent benign rhythms such as mild sinus tachycardia or rate-controlled atrial fibrillation related to our elderly cohort. Non-trauma syncope guidelines recommend telemetry when cardiac etiology is suspected. The suspicion is based on cardiac history, cardiac symptoms, and 12-lead ECG [4]. We recommend this approach for mechanical and syncopal falls alike. Pacemaker placement and revision are much more common for syncopal patients. Screening both groups will identify these patients.

We found equal prevalence of MI in the syncopal and mechanical fall populations (Table 3), which is consistent with previous reports showing that troponins and laboratory tests, in general, are rarely useful for syncope [17, 18]. Our data support the conclusion that routine MI workup is unnecessary for syncopal falls. Cerebrovascular accident (CVA) is not significantly different between the two groups, nor is the condition common overall. Most fall patients with a history of loss of consciousness will undergo brain computed tomography (CT) based on trauma protocols. We believe that it is best to evaluate for stroke based on symptoms rather than a history of syncope. The incidence of recurrent falls is similar, which suggests that evaluation for an underlying condition that could contribute to future falls is equally important for both groups. LOS is not significantly different, demonstrating that the extent of treatment by this measure is not driven by syncopal workup.

Orthostasis was greater for syncopal patients, although not common overall. Testing for orthostasis is easy and low-cost. Orthostasis is often related to medications, which can be adjusted. Other etiologies are hypovolemia due to dehydration and gastrointestinal bleeding, also readily treatable conditions. Because orthostasis is present in both groups and testing is inexpensive and straightforward, we believe it makes sense to check all elderly fall patients for orthostasis.

Our study's short- and long-term death rates are significantly lower for syncopal falls (Tables 2, 4), which differs from the results of Sri-on et al., who found no significant difference in mortality. The number of deaths in their study was extremely low: one in each group for 30-day mortality and three versus six deaths in the mechanical versus syncopal groups at six months [1]. Our larger sample size allowed us to show a difference. The lower syncopal death rate may be because when someone faints, they tend to slump to the floor, whereas mechanical falls may involve greater force. This is supported by the higher incidence of traumatic brain injury in our mechanical group.

Strengths

The large sample size and frequent positive clinical endpoints, including mortality, in the MIMIC ICU registry increase the power to detect differences. An advantage of significant results is that even if we decide that the difference is too small to be clinically meaningful, we can rule out a type 2 error. 

In the fall population, the syncopal episode was severe enough to result in an observable event. This observable sign makes the history more objective than one based only on the patient's recollection of symptoms. The syncopal and mechanical ground-level fall populations can serve as groups matched by a propensity to fall. If a diagnosis is equally common in the syncopal and mechanical fall populations, one may conclude that syncope is not specific for the diagnosis and that the history of syncope should not independently prompt its evaluation.

Limitations

The classification of syncopal versus mechanical falls is based on judgment by the admitting and consulting physicians. Classification also requires judgment on the part of the authors as we abstract the data. This process is inherently subjective, leading to the risk of classification bias. We believe that this bias further limits the accuracy of the syncopal versus mechanical distinction to predict outcome. Imprecision surrounding the mechanism is inherent to retrospective trauma studies, but it also reflects the reality of clinical practice, making our study more pragmatic. Syncopal patients may be subject to more intensive evaluation due to their history, which risks surveillance bias. The ICU population is subject to extensive testing at baseline. Thus, the history of present illness for ICU patients may be less important in driving workup due to the high level of monitoring and evaluation in the ICU.

## Conclusions

After studying patients with ground-level falls classified as syncopal or mechanical in a large, severely ill ICU population, we support the emerging consensus that this classification of etiology adds little to management. Overall, syncopal etiology is of some prognostic value but does not identify a sicker population. Due to similar incidence of cardiovascular conditions, especially the high incidence of arrhythmia in both groups, we recommend considering all elderly falls syncopal by default. Workup should follow standardized guidelines. The only mandatory tests are ECG and orthostatic vital signs; the rest should be guided by symptoms and risk factors. This results in a simple, streamlined algorithm that we can apply to all falls in elderly patients admitted to the ICU, whether syncopal or mechanical. Further study will determine whether we can generalize this algorithm to non-ICU settings.
